# CRISPR/Cas9-mediated knockout of *ZmHMA3* reveals its essential role in zinc homeostasis and high-zinc stress tolerance in maize

**DOI:** 10.1038/s41598-026-53000-w

**Published:** 2026-05-11

**Authors:** Guihua Lv, Youqiang Li, Jianjian Chen, Zhenxing Wu, Wenmei Wu, Xiaohong Wu, Haijian Lin, Tingzhen Wang

**Affiliations:** 1 Institute of Maize and Featured Upland Crops, Zhejiang Academy of Agricultural Sciences, Dongyang, 322100 China; 2 Maize Research Institute, Sichuan Agricultural University, Chengdu, 611130 China

**Keywords:** Maize (*Zea mays*), Heavy metal ATPase (HMA), Zn stress, CRISPR-Cas9 system, Plant biotechnology, Plant ecology, Plant molecular biology, Plant physiology, Plant stress responses

## Abstract

Excessive Zn is toxic to maize (*Zea mays* L.). The heavy metal ATPase gene *ZmHMA3* is associated with heavy metal transport, but its function in maize tolerance to high Zn stress has not been fully characterized. In this study, CRISPR/Cas9 technology was used to generate *zmhma3* knockout mutants to investigate its function under high Zn stress. High Zn stress significantly induced the expression of *ZmHMA3* in maize leaves and roots. Phenotypic analysis showed that, compared to the WT plants, the *zmhma3* mutants exhibited significantly reduced tolerance to excessive Zn, manifested as severe growth inhibition, impaired root structure, decreased activity of key antioxidant enzymes (CAT, POD, SOD), and aggravated membrane damage. Furthermore, the mutants accumulated significantly higher levels of Zn in both roots and leaves, accompanied by disordered subcellular Zn distribution, indicating disrupted intracellular Zn homeostasis. Our results demonstrate that *ZmHMA3* is a key positive regulator in maize’s response to high Zn stress, likely by coordinating Zn compartmentalization and alleviating oxidative damage. This study provides new genetic and physiological insights into the molecular mechanisms of Zn stress tolerance in maize and offers a potential target for breeding new maize varieties tolerant with improved high-Zn-efficiency.

**Supplementary Information:**

The online version contains supplementary material available at 10.1038/s41598-026-53000-w.

## Introduction

Zinc (Zn) is an essential micronutrient for normal plant growth and development, participating in key physiological processes such as enzyme activity regulation, protein stability, and signal transduction^1^. However, excess Zn in soil is highly toxic to plants^2^. Human activities, including mining, industrial emissions, and prolonged application of Zn-containing fertilizers, have elevated Zn levels in some agricultural soils, increasing the risk of Zn overload and toxicity in plants^3^. When Zn accumulation exceeds the tolerance threshold (> 400 µg g^−1^ dry weight), plants exhibit typical toxicity symptoms, including severe inhibition of root growth, leaf chlorosis, and significant reductions in biomass and yield^4,5^. Zn toxicity is prevalent in acidic and sludge-amended soils, which may limit crop yield and quality^6^.

In response to excess Zn in the environment, plants have evolved a complex and finely-tuned homeostatic regulatory network. This network operates coordinately at multiple levels to maintain intracellular Zn ion balance. The cell wall serves as the primary barrier preventing excessive Zn from entering the cytoplasm. Modifications to its components, such as pectin, can significantly alter its Zn-binding capacity of the cell wall for Zn ions, thereby regulating Zn influx into the cell^7,8^. Natural allelic variation in the pectin biosynthesis gene TBR (TRICHOME BIREFRINGENCE) in *Arabidopsis* modulate plant Zn tolerance by altering the degree of pectin methylation in the root cell wall^9^. Intracellularly, free Zn^2+^ can be chelated by metal-binding agents (e.g., phytochelatins and organic acids) or rely on transmembrane transporters for sequestration or efflux^10–13^. Among these, ZIP (Zinc-regulated transporters, Iron-regulated transporter-like Protein) family proteins are primarily responsible for Zn uptake, while members of the MTP (Metal Tolerance Protein)/CDF (Cation Diffusion Facilitator) family, often localized to the tonoplast, sequester excess Zn into the vacuole, constituting a crucial intracellular detoxification strategy^14–17^. The Heavy Metal ATPase (HMA) family occupies a central position in this network^18^. These P-type ATPases utilize ATP hydrolysis to drive the transmembrane transport of divalent cations such as Zn and cadmium (Cd) through ATP hydrolysis, and their functional diversity is closely linked to their subcellular localization. Notably, due to the similar physicochemical properties of Zn and Cd, their transport pathways extensively overlap, and several HMA members have been confirmed to transport both ions^19^. For instance, *Arabidopsis* AtHMA2 and AtHMA4, localized to the plasma membrane of root stele cells, participate in loading Zn and Cd into the xylem, thereby mediating their long-distance transport to the shoots^20,21^. In contrast, AtHMA3 and rice OsHMA3 are localized to the tonoplast. By sequestering Zn^2+^ (and Cd^2+^) into the vacuole, they significantly reduce the concentration of free metal ions in the cytoplasm, thereby enhancing plant tolerance to heavy metals and reducing their translocation to grains^22,23^. Therefore, HMA proteins form a multi-layered regulatory network governing metal uptake, distribution, and detoxification. Furthermore, cross-species comparative genomics and studies of natural variation further reveal the diversity of these tolerance mechanisms. For instance, in Zn hyperaccumulator plants such as *Arabidopsis* halleriand Noccaea caerulescens, the expression levels of genes associated with Zn tolerance and hyperaccumulation (e.g., HMA4, MTP1) are significantly higher than in the model plant *Arabidopsis*^24^. Additionally, linkage analysis and genome-wide association studies (GWAS) have identified multiple quantitative trait loci (QTL) associated with natural variation in high-Zn tolerance in rice, Arabidopsis, and Brassicacrops, highlighting the genetic complexity of this trait^25–27^.

In maize (*Zea mays* L.), our previous genome-wide identification analyses have revealed the presence of HMA family members, with natural variations in *ZmHMA2* and *ZmHMA3* showing significant associations with leaf Cd accumulation, indicating their important roles in the heavy metal regulatory network^18^. Notably, *ZmHMA3* is a key major gene controlling Cd accumulation in maize kernels, and its natural variation is the main cause of the significant differences in kernel Cd content among different maize inbred lines^28^. These studies mainly focus on its core role in Cd detoxification and regulating Cd accumulation in grains. Our recent functional verification further demonstrates that overexpression of *ZmHMA3* can positively regulate maize tolerance to both Cd and Zn^29^. However, existing research approaches-such as natural variation analysis and overexpression-have not yet systematically elucidated the specific physiological function of *ZmHMA3* in maize responses to high-Zn stress at the whole-plant level using loss-of-function mutants. In particular, the core mechanism by which *ZmHMA3* mediates high-Zn tolerance, especially through the regulation of Zn subcellular distribution, remains a critical knowledge gap.

To elucidate the specific function of *ZmHMA3* in maize response to Zn toxicity, this study generated *ZmHMA3* loss-of-function mutants using CRISPR/Cas9 technology and systematically compared the phenotypic and physiological responses of *zmhma3* mutants and wild-type (WT) plants under high-Zn stress. The results demonstrated that the loss of *ZmHMA3* significantly reduced high-Zn tolerance in maize, manifested as inhibited plant growth, impaired root development, decreased antioxidant enzyme activity, reduced cell membrane stability, excessive Zn accumulation and altered translocation, as well as disrupted subcellular compartmentalization. These findings collectively indicate that *ZmHMA3* plays a crucial role in maintaining Zn homeostasis, mitigating oxidative damage, and promoting growth, thereby regulating maize tolerance to high-Zn stress. This study not only provides direct evidence at the physiological and cellular levels for the function of *ZmHMA3* in regulating high-Zn tolerance in maize, but also offers new genetic insights into the molecular mechanisms of Zn homeostasis regulation in maize, serving as a potential target for crop stress resistance improvement through key gene manipulation.

## Results

### Expression characteristics under high-Zn stress of *ZmHMA3* in maize

**Fig. 1:**
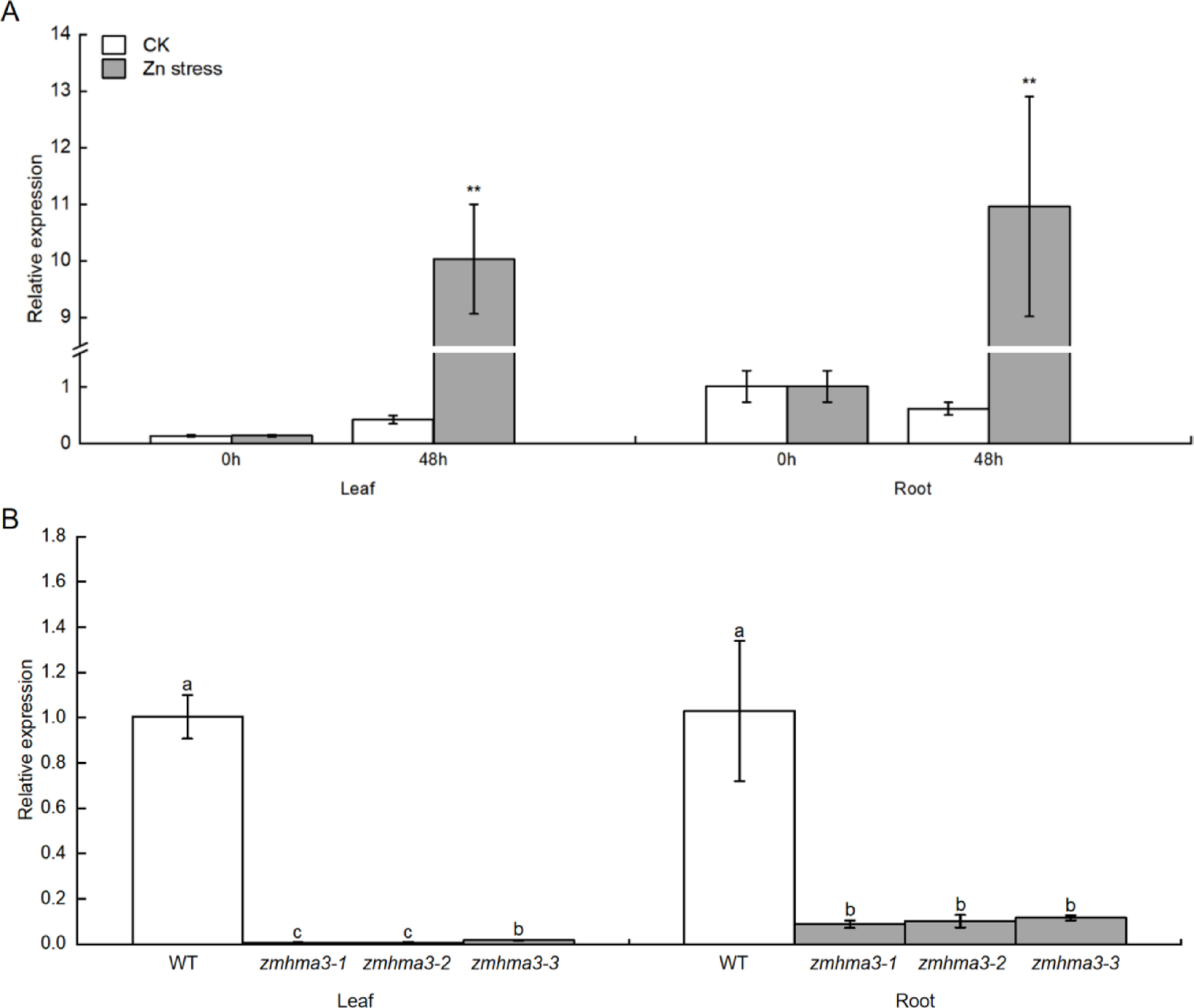
The expression pattern of *ZmHMA3* under Zn stress*.* (**A**) Relative expression of *ZmHMA3* in leaves and roots of B104 maize under 0 and 48 h high-Zn stress. (**B**) Relative expression of z*mhma3* homozygous mutant lines and WT in leaves and roots under 48 h high-Zn stress. *ZmActin* was employed as the internal reference gene. Data are presented as mean ± *SE* (*n* = 3). The standard error is indicated. The ** and different letters indicate significant differences determined by Student’s *t*-test and one-way ANOVA followed by Duncan’s test. Different letters correspond to *P* < 0.05, * stands corresponds to *P* < 0.05, and ** corresponds to *P* < 0.01.

In the previous association analysis study, we reported that overexpression of *ZmHMA3* could promote the accumulation of Zn and Cd in maize, suggesting its potential role in positively regulating high-Zn tolerance^15^. To systematically verify the gene function of *ZmHMA3* under high-Zn conditions, we first investigated the expression changes of *ZmHMA3* in different tissues at various time points after high-Zn treatment using qRT-PCR. The results showed that after 48 h of high-Zn stress, the expression of *ZmHMA3* was significantly up-regulated in both maize leaves and roots (Fig. 1A). This indicates that high-Zn stress can induce the expression of *ZmHMA3*, suggesting that *ZmHMA3* may be involved in the tolerance response of maize to high-Zn stress.

### CRISPR/Cas9-mediated knock-out of *ZmHMA3* decreased tolerance in high-Zn stress

**Fig. 2:**
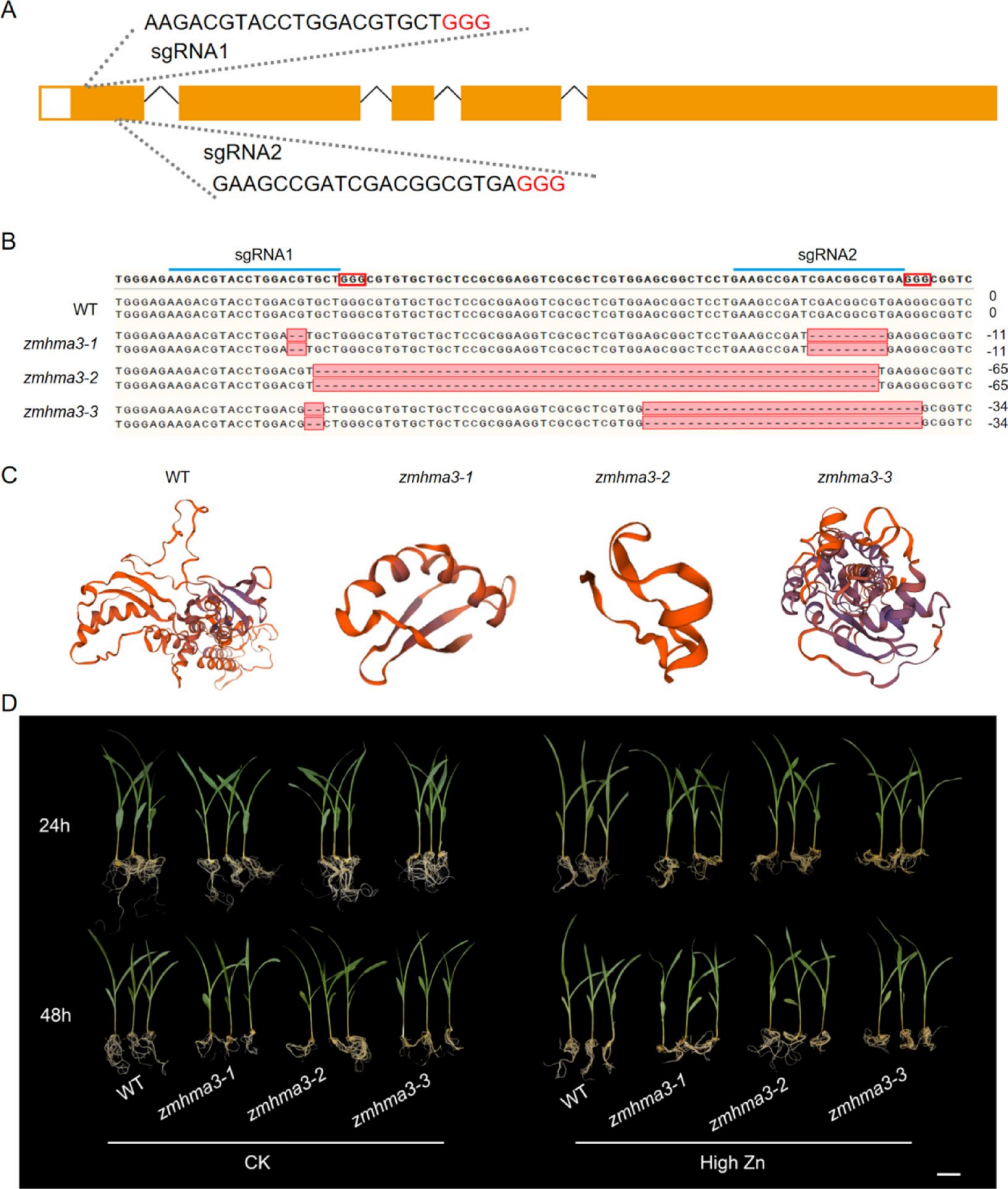
CRISPR/Cas9 system and knock-out phenotype of *zmhma3* mutants. (**A**) Schematic diagram of CRISPR/cas9 target site in *ZmHMA3* gene. Fold lines represent introns and orange boxes represent exons and PAMS (NGGs or CCNs) are marked in red. (**B**) Mutation site in z*mhma3* homozygous mutant lines. (**C**) Amino acid conformational mutations caused by base frameshift mutation. (**D**) The WT and *zmhma3* mutant plants under high-Zn stress for 24 and 48 h. Bar = 2 cm.

To investigate the functional role of *ZmHMA3*, we generated *zmhma3* mutant lines via the CRISPR/Cas9 genome-editing approach, in which the *ZmHMA3* expression was substantially reduced or absent (Fig. 1B). The Cas9/sgRNA construct contained a synthetic sgRNA targeting the first and second exons of *ZmHMA3* (Fig. 2A). Following confirmation by PCR and sequencing, multiple chimeric T_0_ plants carrying various frameshift insertion or deletion alleles were obtained in maize. These T_0_ plants were self-pollinated to produce T_1_ seeds, which were subsequently genotyped. Through Sanger sequencing analysis and T_2_ embryo-endosperm section screening, three Cas9-free homozygous transgenic lines (*zmhma3-1*, *zmhma3-2*, and *zmhma3-3*) were obtained for phenotypic characterization. Their genomic sequences carried frameshift mutations that led to alterations in the ZmHMA3 protein sequence and conformation (Fig. 2B and C). Both wild type (WT) and *zmhma3* mutant plants were subjected to high-Zn stress for specific durations (24 and 48 h). Phenotypic observations showed that after 48 h of high-Zn stress, both WT and *zmhma3* mutant plants exhibited wilting. However, the wilting was more pronounced in the *zmhma3* mutant plants compared to WT plants (Fig. 2D).

### *ZmHMA3* affects the seedling growth vigor and leaf cell membrane permeability of maize under high-Zn stress

**Fig. 3:**
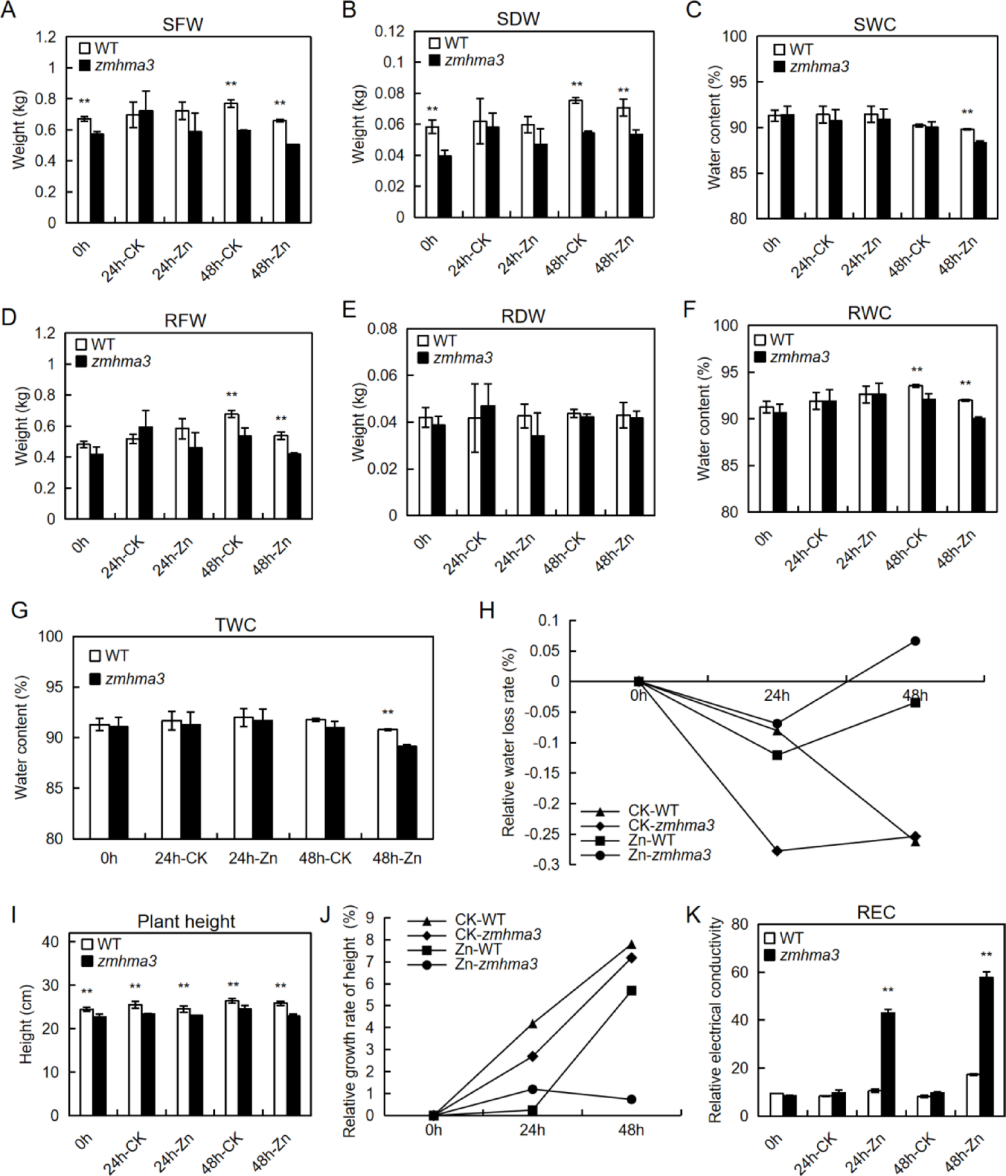
Effects of *ZmHMA3* loss on water-related traits, plant height, and cell membrane permeability in maize under high-Zn stress. (**A**) Shoot fresh weight (SFW), (**B**) shoot dry weight (SDW), and (**C**) shoot water content (SWC) of WT and *zmhma3* mutant plants at 0, 24, and 48 h after high-Zn stress treatment (*n* = 12). (**D**) Root fresh weight (RFW), (E) root dry weight (RDW), and (**F**) root water content (RWC) of WT and *zmhma3* mutant plants at 0, 24, and 48 h after high-Zn stress treatment (*n* = 12). (**G**) Total water content (TWC) of WT and *zmhma3* mutant plants at 0, 24, and 48 h after high-Zn stress treatment (*n* = 12). (**H**) Relative water loss rate of WT and *zmhma3* mutant plants at 0, 24, and 48 h after high-Zn stress treatment. Relative water loss rate = (post treatment water content − initial water content)/initial water content × 100%, where the initial water content was obtained from the 0-h treatment. (**I**) The plant height of WT and *zmhma3* mutant plants at 0, 24, and 48 h after high-Zn stress treatment (*n* = 15). (**J**) Relative plant height growth rate of WT and *zmhma3* mutant plants at 0, 24, and 48 h after high-Zn stress treatment. Relative plant height growth rate = (post treatment plant height − initial plant height)/initial plant height × 100%, where the initial plant height was obtained from the 0-h treatment. (**K**) Relative conductivity of WT and *zmhma3* mutant plants at 0, 24, and 48 h after high-Zn stress treatment (*n* = 15). Data are presented as mean ± *SE*. The standard error is indicated. The * indicates significant difference determined by Student’s *t*-test. * stands corresponds to *P* < 0.05, and ** corresponds to *P* < 0.01.

To further investigate the gene function of *ZmHMA3*, we measured a set of physiological parameters in WT and *zmhma3* mutants under high-Zn stress, including seedling fresh weight (SFW), seedling dry weight (SDW), seedling water content (SWC), root fresh weight (RFW), root dry weight (RDW), root water content (RWC), total water content (TWC), plant height, and Relative electrical conductivity (REC). The results indicated that after 48 h of high-Zn stress, *zmhma3* mutants exhibited significant growth inhibition compared to the WT. Specifically, after 24 h of stress, no significant differences were observed between WT and *zmhma3* mutants in fresh weight (SFW, RFW), dry weight (SDW, RDW), or water content (SWC, RWC, TWC). However, after 48 h of stress, the *zmhma3* mutants showed significant decreases in SFW, SDW, SWC, RFW, and RWC (Fig. 3A–F). Although RDW did not differ significantly between genotypes, TWC of *zmhma3* mutants remained lower after 48 h of high-Zn stress (Fig. 3G). Based on the 0 h baseline, the relative water loss rate was most pronounced in Zn-stressed *zmhma3* mutants (Fig. 3H), a trend consistently observed at the shoot, root, and whole-plant levels (Fig. S1), indicating that knockout of *ZmHMA3* exacerbated the reduction in fresh weight and water content under high-Zn stress.

Since the plant height of *zmhma3* mutants was consistently lower than that of WT (Fig. 1I), we further compared the plant height growth rate using the 0 h value as the baseline. The results indicated that after 48 h of high-Zn stress, the elongation rate of *zmhma3* mutants was significantly lower than that of WT, suggesting that loss of *ZmHMA3* enhances the suppression of plant growth under high-Zn conditions (Fig. 1J). Moreover, after 24 h and 48 h of high-Zn treatment, the relative electrical conductivity (REC) increased more rapidly in *zmhma3* mutants and was significantly higher than in WT, implying more severe membrane damage (Fig. 1K).

It should be noted that baseline differences in certain traits (e.g., SFW, SDW, plant height) were already present at 0 h between WT and *zmhma3* mutants, indicating that loss of *ZmHMA3* may affect normal plant development. To exclude the interference of such developmental differences on the stress response analysis, we performed an analysis of covariance (ANCOVA) using the 0 h measurements as covariates. After correction, high-Zn stress still caused significantly lower SFW in *zmhma3* mutants compared with WT. Although SDW and plant height did not reach significance in the ANCOVA, *zmhma3* mutants showed a greater downward trend in both relative water loss rate and relative plant height growth rate (Table S1). In summary, loss of *ZmHMA3* under high-Zn stress led to significantly reduced water retention, decreased growth rate, and aggravated membrane damage in maize, supporting the important role of *ZmHMA3* in maize tolerance to high-Zn stress.

### *ZmHMA3* affects the root morphology of maize under high-Zn stress

**Fig. 4:**
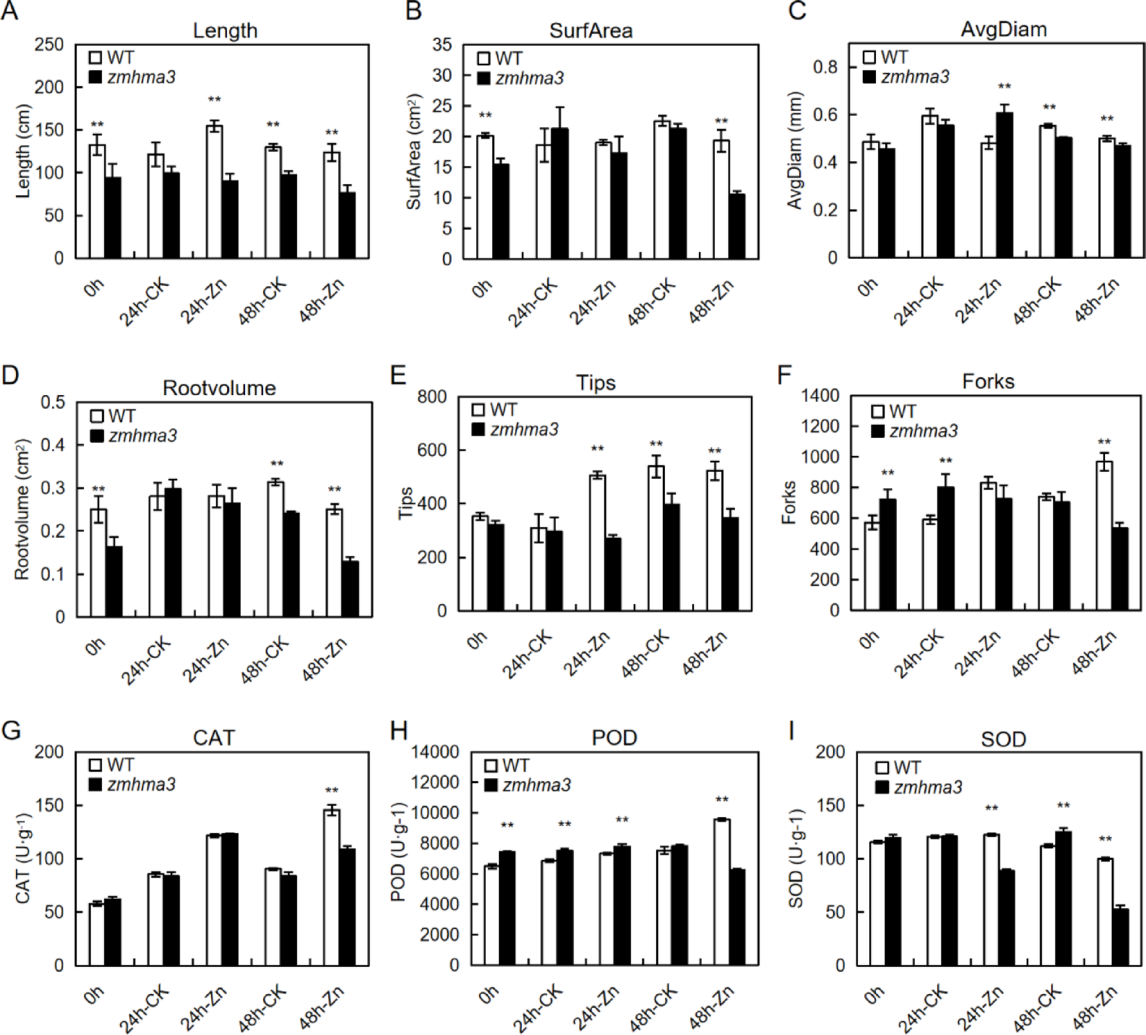
Effects of *ZmHMA3* on root morphological traits and antioxidant enzyme activities in maize under high-Zn stress. (**A**) Total root length. (**B**) Root surface area (SurfArea). (**C**) Root diameter (AvgDiam). (**D**) Root volume. (**E**) Tips. (**F**) Forks of WT and *zmhma3* mutant plants at 0, 24, and 48 h after high-Zn stress treatment (*n* = 15). of (**G**) Catalase (CAT), (**H**) peroxidase (POD), and (**I**) superoxide dismutase (SOD) activities in WT and *zmhma3* mutant plants at 0, 24, and 48 h after high-Zn stress treatment (*n* = 15). Data are presented as mean ± *SE*. The standard error is indicated. The * indicates significant difference determined by Student’s *t*-test. * Stands corresponds to *P* < 0.05, and ** corresponds to *P* < 0.01.

To elucidate the role of *ZmHMA3* in root responses to high-Zn stress, we analyzed root morphological traits in WT and *zmhma3* mutants. Under high-Zn stress, the *zmhma3* mutants exhibited significant suppression of root growth and structural damage compared to WT plants. Under control conditions, all root traits of WT plants showed an increasing trend except for root length, while most traits of the *zmhma3* mutants also increased, though the root length and fork number displayed an initial rise followed by a decline. Under high-Zn stress, WT plants maintained stable root length, surface area, diameter, and volume, while root tip number and fork number increased significantly. In contrast, the *zmhma3* mutants showed a distinct response pattern, with root surface area, diameter, volume, and fork number increasing initially and then decreasing, whereas root tip number exhibited the opposite trend (Fig. 4A–F).

Most notably, after 48 h of high-Zn stress treatment, *zmhma3* mutants displayed significantly lower values across all measured root parameters-including root length, surface area, average diameter, root volume, tip number, and fork number—compared to WT plants (Fig. 4A–F). Although WT plants showed superior performance in root length, surface area, and volume prior to stress treatment, indicating inherent differences in basal root development (Fig. 4A,B, and D), ANCOVA with 0 h data as a covariate confirmed that these three parameters in *zmhma3* mutants remained significantly lower than in WT plants after 48 of stress (Table S1). These findings demonstrate that loss of *ZmHMA3* function enhances the sensitivity of maize roots to high-Zn stress, leading to more severe inhibition of root growth and structural impairment.

### *ZmHMA3* affects antioxidant defense response to high-Zn stress in maize

To elucidate the antioxidative response mechanism of maize roots to high-Zn stress, we analyzed the changes in antioxidant enzyme activities in WT plants and *zmhma3* mutants under high-Zn stress at 24 and 48 h. The results showed that under 24 h stress, the catalase (CAT) activity of both WT plants and *zmhma3* mutants increased significantly, with no significant difference between them. However, after 48 h of stress, CAT activity in WT plants continued to rise and was significantly higher than that in *zmhma3* mutants (Fig. 4G). In terms of peroxidase (POD) activity, the POD activity of *zmhma3* mutants was significantly higher than that of WT at 24 h of stress, but it decreased significantly and fell below WT levels at 48 h (Fig. 4H). For superoxide dismutase (SOD), the SOD activity of WT plants was already significantly higher than that of *zmhma3* mutants at 24 h of stress, and this difference further widened at 48 h (Fig. 4I). Collectively, these results indicate that the activities of all three major antioxidant enzymes in *zmhma3* mutants were significantly lower than those in WT plants during the later stage (48 h) of high-Zn stress, suggesting that the loss of *ZmHMA3* gene function leads to a systemic impairment of antioxidative capacity in maize under high-Zn stress.

### *ZmHMA3* affects Zn homeostasis and subcellular compartmentalization in maize under high-Zn stress

**Fig. 5:**
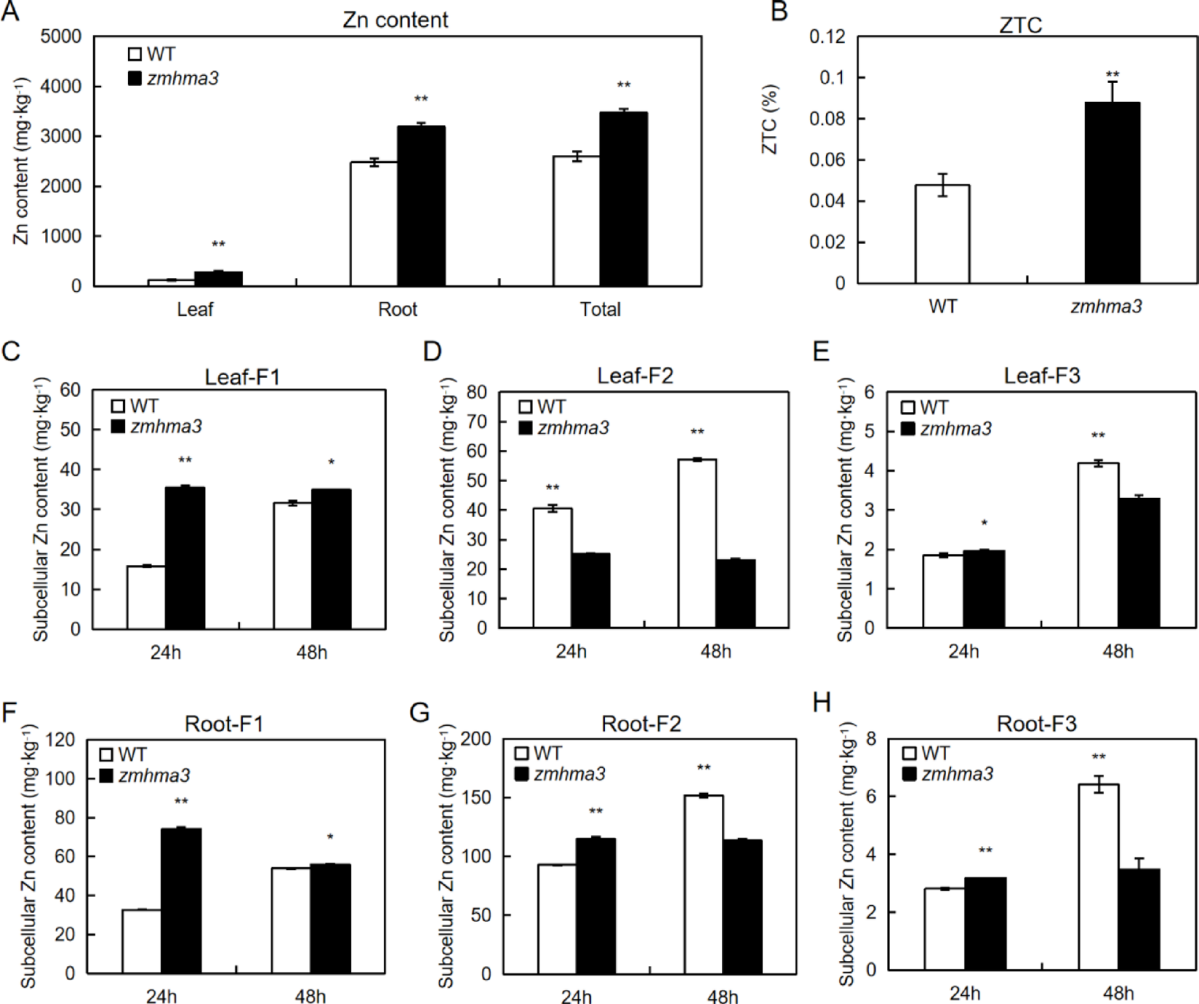
Effects of *ZmHMA3* on tissue-specific accumulation, subcellular distribution, and transport coefficient of Zn content in maize under high-Zn stress. (**A**) Zn content in leaves, roots, and whole plants of WT and *zmhma3* mutant plants at 48 h after high-Zn stress treatment (*n* = 15). (**B**) Zn transport coefficient (ZTC) of WT and *zmhma3* mutant plants at 48 h after high-Zn stress treatment (*n* = 15). ZTC = leaf Zn content/root Zn content. (**C**) Cell wall fraction (F1), (**D**) soluble fraction (F2), and (**E**) cell membranes and organelles fraction (F3) in leaves of WT and *zmhma3* mutant plants at 24 and 48 h after high-Zn stress treatment (*n* = 20), (**F**) F1 fraction, (**G**) F2 fraction, and (**H**) F3 fraction in roots of WT and *zmhma3* mutant plants at 24 and 48 h after high-Zn stress treatment (*n* = 20). Data are presented as mean ± *SE*. The standard error is indicated. The * indicates significant difference determined by Student’s *t*-test. * stands corresponds to *P* < 0.05, and ** corresponds to *P* < 0.01.

To investigate the role of *ZmHMA3* in Zn accumulation and compartmentalization in maize under high-Zn stress, we measured tissue-specific Zn accumulation, subcellular distribution, and the Zn transport coefficient (leaf Zn content/root Zn content, ZTC) in WT plants and *zmhma3* mutants. The results showed that after 48 h of high-Zn stress, Zn content in leaves, roots, and the whole plant was significantly higher in *zmhma3* mutants than in WT plants, with root Zn content significantly exceeding leaf Zn content (Fig. 5A), indicating that loss of *ZmHMA3* function leads to excessive Zn accumulation in both aerial and underground tissues. Analysis of the ZTC revealed that the *zmhma3* mutants had a significantly higher ZTC than WT plants, suggesting an altered efficiency of Zn translocation from roots to shoots (Fig. 5B).

Subcellular Zn distribution analysis demonstrated that in leaves, Zn content in the F1 fraction (cell wall) of *zmhma3* mutants was significantly higher than that of WT plants at 24 h but decreased to a level similar to WT plants at 48 h (Fig. 5C). In the F2 fraction (soluble fraction, including cell sap and cytosol), Zn content in *zmhma3* mutants were significantly lower than that in WT plants at both 24 h and 48 h (Fig. 5D). In the F3 fraction (cell membranes and organelles), Zn content in *zmhma3* mutants were significantly lower than that in WT plants at 24 h and remained relatively low at 48 h, though the difference diminished (Fig. 5E).

In roots, Zn content in the F1 fraction of *zmhma3* mutants were significantly higher than that of WT plants at 24 h but decreased to a level comparable to WT plants at 48 h (Fig. 5F). In the F2 and F3 fractions, Zn content in *zmhma3* mutants was higher than in WT plants at 24 h but lower at 48 h (Fig. 5G and H). Throughout the stress period, Zn content in all subcellular fractions of WT plants gradually increased, whereas in *zmhma3* mutants, Zn content in the leaf F1 fraction showed no significant change, decreased significantly in F2, and increased significantly in F3. In roots, Zn content in the F1 fraction of *zmhma3* mutants decreased significantly, while no significant changes were observed in the F2 and F3 fractions (Fig. 5C–H).

These subcellular distribution results indicate that the loss of *ZmHMA3* disrupts Zn compartmentalization within cells, potentially impairing the normal translocation of Zn from the cell wall to the intracellular space and leading to compartmentalization disorder. In summary, loss of *ZmHMA3* function results in excessive Zn accumulation in roots and leaves, aberrant Zn translocation, and disrupted subcellular Zn distribution in maize under high-Zn stress.

## Discussion

Maintaining intracellular ion homeostasis is crucial for normal plant growth and development^30^. *ZmHMA3* plays a complex and finely tuned regulatory role in Zn ion homeostasis. High-Zn stress significantly induces the expression of *ZmHMA3* in roots and leaves (Fig. 1A), a pattern similar to that of known positive regulators of Zn homeostasis such as *OsHMA2*, *AtHMA2*, and *HvHMA2*, suggesting it may perform a similar function^21,31,32^. Notably, *ZmHMA3* is localized to the plasma membrane^29^, and *zmhma3* mutants exhibit excessive Zn accumulation in both roots and leaves (Fig. 5A). This phenotype closely matches the characteristics observed when plasma membrane-localized transporters responsible for Zn efflux (such as *Arabidopsis AtHMA2*) lose their function^21^. However, this appears to superficially contradict our previous finding that overexpression of *ZmHMA3* simultaneously enhances maize high-Zn tolerance and promotes Zn accumulation^29^.

Previous studies indicate that the key determinants of tolerance are the chemical speciation of Zn ions and their spatial distribution within the cell^33–35^. Accordingly, we propose that plasma membrane-localized *ZmHMA3* may primarily regulate the pattern and efficiency of Zn influx rather than determining net Zn accumulation. In overexpression plants, the high abundance of *ZmHMA3* may act as an efficient ‘Zn^2+^ influx pump’, mediating a controlled, substantial influx of Zn^36,37^. This enhanced influx acts as a strong physiological signal that efficiently activates the downstream detoxification program, including metal chelators synthesis (such as phytochelatins)^38^ and upregulation of tonoplast-localized transporters (such as MTPs, ZIPs)^39,40^, facilitating Zn sequestration into the vacuole for storage. Thus, the observed elevated Zn in the cell wall (F1) and cytosol (F2) in overexpression plants reflects a virtuous cycle of ‘high influx—high detoxification’, achieving high accumulation with tolerance^29^. This is essentially consistent with the tolerance mechanism of natural Zn hyperaccumulator plants^41,42^.

Conversely, *zmhma3* mutants lack this regulatory influx pump. Zn enters cells passively and disorganized, failing to trigger effective detoxification and leading to uncontrolled intracellular distribution^43^. The subcellular distribution data from this study provide direct evidence for this. The significantly lower cytosolic Zn (F2) in mutants is likely due to impaired chelation/transport, causing Zn to persist in toxic forms and induce oxidative stress (Fig. 5D,E,G, and H)^44^. The temporary increase in cell wall (F1) Zn content at an early stage (Fig. 5C and F) may represent a rapid but saturable compensatory response^7^. Thus, the mutant’s higher total Zn represents pathological accumulation from disordered influx and detoxification failure. It is noteworthy that the Zn content in overexpression plants is even higher than in the mutant (Fig. 5A), which further supports the hypothesis that the ‘pattern of influx’ rather than the ‘amount of influx’ is the key difference. *ZmHMA3* likely functions as a key positive regulator in maize under high-Zn stress by mediating Zn^2+^ influx across the plasma membrane, thereby orchestrating the activation of downstream detoxification pathways and Zn compartmentalization. However, further experiments (such as fluorescence protein labeling and yeast heterologous system assays) are still needed to accurately analyze the subcellular distribution of Zn, and the downstream detoxification mechanisms require elucidation to clarify the regulatory network of *ZmHMA3*, thereby enabling a more comprehensive understanding of the maize Zn homeostasis mechanism.

Besides intracellular compartmentalization, HMA family proteins are also involved in the inter-tissue distribution of metals. In *Arabidopsis*, *AtHMA2* and *AtHMA4* are responsible for loading Zn (along with Cd and Pb) into the xylem, thereby promoting its translocation to the shoots. In contrast, *AtHMA3* reduces the entry of these metals into the xylem stream directed toward the shoots through vacuolar sequestration. In this study, it was observed that the ZTC of the *zmhma3* mutants was significantly higher than that of the wild type (Fig. 5B), similar to the case with *AtHMA3*. However, considering that the ZTC of *ZmHMA3*-overexpressing plants also increased significantly, the elevated ZTC in *zmhma3* mutants may also stem from disrupted Zn compartmentalization and transport dysregulation, which requires further validation in future studies. A key and unresolved question is how this altered distribution affects the Zn content in reproductive organs, particularly in the grains.

In agricultural production, crops in high-Zn soils need to be tolerant while keeping grain Zn within a safe range (≤ 50 mg/kg). Studies on HMA genes in other species offer insights. In rice, overexpressing *OsHMA3* lowers grain Cd without affecting Zn^23^, whereas knocking out *OsHMA2* reduces both Zn and Cd in upper tissues and harms growth^45^. In *Arabidopsis*, variation in *HMA3* mainly alters Cd distribution, with little effect on leaf Zn, hinting at robust Zn homeostasis^37^. Interestingly, in sweet corn, loci controlling grain Cd do not affect Zn content^46^. This suggests the potential to breed crops that tolerate high Zn while maintaining grain Zn nutrition. Our study focused on seedlings; how *ZmHMA3* affects Zn distribution to grains during reproduction requires urgent future research to assess its full breeding potential.

Disruption of Zn homeostasis and subsequent oxidative damage jointly heighten the sensitivity of the *zmhma3* mutants to high-Zn stress. Heavy metal stress triggers excessive ROS production in plants, causing oxidative damage to biomolecules and membranes^47,48^. Plants counteract this through a sophisticated antioxidant system^49,50^. In the Zn hyperaccumulator *Sedum alfredii*, the activities of SOD, CAT, and GPX increase under high-Zn stress and exceed those of non-hyperaccumulating ecotypes^51^. In this study, the activities of SOD, CAT, and POD in the mutant were significantly lower than in the WT, indicating a compromised antioxidant defense (Fig. 4G–I). This decline in antioxidant function correlated with severe physiological disturbances: decreased fresh/dry weight and water content, elevated water loss, and increased membrane permeability (Fig. 3)^52^. The combined direct toxicity of Zn (e.g., mitochondrial disruption, enzyme inhibition)^53,54^ and secondary oxidative injury (ROS-induced lipid peroxidation and macromolecular damage)^2,55^ collectively undermined the mutant’s high-Zn-stress tolerance. Notably, as the primary organ exposed to soil high-Zn stress, the roots show markedly impaired morphology in the *zmhma3* mutant. They display significantly reduced root length, surface area, diameter, volume, tip number, and forks (Fig. 4A–F)—a phenotype aligning with reported Zn toxicity symptoms in other species^56,57^. In *Arabidopsis*, Zn stress even disrupts root hair morphology, likely through impairing root hair-associated proteins like ROOT HAIR DEFECTIVE 3 (RHD3) and ROOT HAIR MORPHOGENESIS 5 (MRH5/SHV3)^58^. Furthermore, this root growth limitation compromises water and nutrient uptake, ultimately restricting shoot development^59^. Thus, loss of *ZmHMA3* disrupts Zn homeostasis, antioxidant protection and plant (especially root) growth, synergistically heightening sensitivity to high-Zn stress.

In summary, this study establishes that *ZmHMA3* is a key positive regulator of maize tolerance to high-Zn stress. Loss of its function severely compromises high-Zn tolerance, leading to a syndrome of symptoms including growth inhibition, root impairment, membrane damage, collapse of the antioxidant system, and dysregulated Zn accumulation and subcellular distribution. These findings demonstrate that *ZmHMA3* is indispensable for maintaining Zn homeostasis and mitigating oxidative damage under Zn toxicity. This work defines the physiological role of *ZmHMA3* at the seedling stage, advancing our understanding of Zn homeostasis in maize. However, this study has certain limitations. It focuses on short-term seedling responses, and the impact of *ZmHMA3* on the full life cycle (especially on grain Zn content and yield) remains unknown, which is critical for assessing its breeding value. Additionally, the precise biochemical activity and transport direction of *ZmHMA3* towards Zn require further validation. Future research should prioritize elucidating how *ZmHMA3* coordinates environmental stress tolerance and grain nutritional safety, to build a stronger foundation for the genetic improvement of stress-resistant maize.

## Materials and methods

### Plant materials and high-Zn stress treatment

The plant materials used in this study were the maize (*Zea mays* L.) inbred line B104. B104 seeds were first washed thoroughly with deionized water, surface-sterilized by shaking in 20% H_2_O_2_ for 20 min, and then rinsed 4–6 times with deionized water (3 min per rinse). The sterilized seeds were soaked overnight in saturated CaSO_4_ solution to promote uniform germination. The following day, the seeds were placed on germination paper and grown in an artificial climate chamber. When seedlings reached the two-leaf (V2) stage, the endosperm was removed, and the plants were transferred to a half-strength nutrient solution for 2 days. Thereafter, the seedlings were randomly divided into two groups: the first group served as the control, and the second group was subjected to high-Zn stress (800 μmol/L ZnSO_4_·7H_2_O). Both groups were cultured in a full‑strength nutrient solution with continuous aeration throughout the experiment.

To obtain *zmhma3* mutants, CRISPR/Cas9 genome editing technology was employed following the previously described method^60^. Briefly, two single-guide RNAs (sgRNAs) targeting the first exon of the *ZmHMA3* gene were designed using the online tool CRISPR-GE (http://skl.scau.edu.cn/). The synthesized sgRNA sequences were ligated into the CRISPR/Cas9 vector linearized by double digestion with SacI and EcoRI. Subsequently, the constructed vector was introduced into *Agrobacterium tumefaciens* strain EHA105, and B104 maize plants were transformed via *Agrobacterium*-mediated transformation, as reported previously^61^. Homozygous T_3_ mutant seeds were obtained through endosperm cutting, Sanger sequencing, and qRT-PCR analysis. These homozygous *zmhma3* mutants were used as experimental materials in subsequent studies.

Plants were sampled at 0 h, 24 h, and 48 h for phenotypic observation, determination of relevant physiological and biochemical parameters, and analysis of Zn content. Three biological replicates were established, each consisting of tissue pooled from four to five plants^29^.

### RNA isolation and qRT-PCR analysis

To investigate the expression pattern of *ZmHMA3* under high-Zn stress, roots and leaves of B104 seedlings were sampled and analyzed at 0 h and 48 h after stress treatment. Additionally, to examine the changes in *ZmHMA3* expression in *zmhma3* mutants, roots and leaves of seedlings from each mutant line were collected and measured after 48 h of high-Zn stress. Three biological replicates were established, each consisting of tissue pooled from three plants.

Total RNA was extracted using TRIzol reagent (Invitrogen, Gaithersburg, MD, USA), and first-strand cDNA synthesis was performed using the PrimeScript™ RT 1^st^ Strand cDNA Synthesis kit (Code Ds10A, TaKaRa, Kyoto, Japan). Subsequently, the qRT-PCR amplification was performed on a CFX96 system (Bio-Rad, Hercules, CA, USA) with TB Green® Premix Ex Taq™ II (Takara, Kyoto, Japan) under the following cycling program: 95 °C for 30 s, followed by 39 cycles at 95 °C for 5 s, 60 °C for 30 s, and then 95 °C for 10 s^29^. The relative expression level of *ZmHMA3*, with three technical replicates for each sample, was determined using the 2^-ΔΔCT^ method^38^. All primers employed in this study are listed in Supplementary Table S2.

### Agronomic trait analysis

For water-related traits, aerial and underground parts of WT and *zmhma3* mutant plants were collected at 0, 24, and 48 h under high-Zn stress. Fresh weights of shoots (SFW) and roots (RFW) were measured immediately after sampling. Samples were then killed at 80 °C for 0.5 h and dried to constant weight at 65 °C to determine shoot dry weight (SDW) and root dry weight (RDW). Tissue water content was calculated as (fresh weight − dry weight)/fresh weight × 100%. To quantify changes in plant water balance under stress, the relative reduction rate of water content was further calculated as (post treatment water content − initial water content)/initial water content × 100%, where the initial water content was obtained from the 0 h treatment^29^. Plant height was measured with a ruler from the stem base to the highest naturally extended leaf tip. Three biological replicates were set up, each consisting of four to five seedlings. All sampling was performed between 9:00 and 10:00 a.m. to minimize diurnal variation-induced errors.

### Relative conductivity analysis

At 0, 24, and 48 h of high-Zn stress treatment, 0.3 g of leaves of similar size (W) from WT and *zmhma3* mutant plants were collected, washed, and gently blotted dry. The samples were placed in 50 mL tubes containing 30 mL deionized water and shaken at 40 rpm in the dark at room temperature for 5 h. The conductivity of the leaching solution (R1) was then measured using a conductivity meter. Subsequently, the tubes were incubated in a 95 °C water bath for 1 h, cooled to room temperature, and mixed thoroughly before the conductivity was measured again (R2). Relative conductivity was calculated as: R1/R2/W × 100%^62^. Three biological replicates were established, with each replicate consisting of five seedlings. All sampling was conducted between 9:00 and 10:00 a.m. to minimize errors induced by diurnal variation.

### Root morphology analysis

Root morphological parameters were measured using a high-throughput image analysis system. Fresh root samples from WT plants and *zmhma3* mutants were collected at 0, 24, and 48 h after high-Zn stress treatment. Roots were spread in deionized water to minimize overlap and then scanned with an Epson Expression 12000XL scanner at 600 dpi. The scanned images were analyzed with TAIR Root Analysis Software to automatically quantify total root length, surface area, average diameter, and number of root tips. Spatial calibration was performed with a standard scale before analysis to ensure measurement accuracy. During root arrangement, primary lateral roots were spread as much as possible to avoid crossing and overlapping. The experiment included three biological replicates, each consisting of five seedlings^63^.

### Antioxidant enzyme activity analysis

Root samples from WT and *zmhma3* mutant plants were collected at 0, 24, and 48 h of high-Zn stress treatment. After rinsing with deionized water and blotting dry, tissues were flash-frozen in liquid nitrogen and ground into fine powder. Approximately 0.1 g of leaf or root powder was used for activity assays of superoxide dismutase (SOD, BC0175), peroxidase (POD, BC0095), and catalase (CAT, BC0205) with corresponding biochemical kits (Solarbio, Beijing, China) using a microplate reader (Multiskan SkyHigh, Thermo Fisher, USA). The experiment included three biological replicates, each consisting of five seedlings. All sampling was performed between 9:00 and 10:00 a.m. to minimize errors caused by diurnal variation.

### Determination of Zn content

To measure the Zn ion content, shoots and roots of both WT and *zmhma3* mutant plants were sampled at 0, 24, and 48 h after high-Zn stress treatment. Then 0.2 g of dry samples was digested with 10 mL of HNO_3_-H_2_O_2_ (5:1, v/v). The concentration of elements in the digestion solution was then determined using inductively coupled plasma mass spectrometry (ICP-MS 7700 X), following a previously established method^64^. The Zn transport coefficient (ZTC) was calculated as leaf Zn content/root Zn content. The experiment included three biological replicates, each consisting of five seedlings. All sampling was performed between 9:00 and 10:00 a.m. to minimize errors caused by diurnal variation.

Subcellular fractionation was performed using a modified differential centrifugation method. Shoots and roots of both WT and *zmhma3* mutant plants were sampled at 0, 24, and 48 h after high-Zn stress treatment. Fresh tissue (1.0 g) was homogenized in 10 mL of ice-cold extraction buffer (containing 250 mmol L^−1^ sucrose, 50 mmol L^−1^ Tris HCl (pH 7.5), and 1 mmol L^−1^ DTT). All subsequent steps were carried out at 4 °C. The homogenate was first centrifuged at 3000 *g* for 15 min at 4 °C, and the resulting pellet was collected as the cell-wall fraction (F1). The supernatant was further centrifuged at 12,000 *g* for 45 min at 4 °C to yield the membrane- and organelle-enriched pellet (F3) and the soluble component/cytosol and cytoplasm component (supernatant, F2). All fractions were digested with a mixture of HNO_3_-H_2_O_2_ (5:1, v/v), and Zn concentrations were determined by atomic absorption spectroscopy^29^. We acknowledge that this method may involve some degree of cross-contamination between fractions. To minimize cross-contamination, the pellets obtained after each centrifugation step were gently washed once with a small volume of ice-cold extraction buffer, and the washings were combined with the corresponding supernatant. To reduce experimental error and improve reliability, four biological replicates were included for each treatment, with each replicate consisting of pooled tissues from five individual plants.

### Data analysis

Statistical analyses were performed using one-way ANOVA followed by Duncan’s multiple-range test (*p* < 0.05) for comparisons among multiple groups, with Student’s *t*-test applied for pairwise comparisons. To account for inherent developmental differences between genotypes, analysis of covariance (ANCOVA) was employed using 0-h measurements as covariates, and the *Bonferroni* correction was applied for significance testing. Data visualization and graph construction were carried out in OriginPro 2021 (OriginLab, USA) and Microsoft Excel (Microsoft, USA). All figure panels were assembled and formatted using Adobe Photoshop 2022 (Adobe Inc., USA) and Microsoft PowerPoint. All experiments included three independent biological replicates.

## Supplementary Information

Below is the link to the electronic supplementary material.

Supplementary Material 1

Supplementary Material 2

Supplementary Material 3

## Data Availability

All data generated or analyzed during this study are included in this published article.
