# Supplementary figures and images for: CRISPR/Cas9-mediated knockout of ZmHMA3 reveals its essential role in zinc homeostasis and high-zinc stress tolerance in maize
Source: Sci Rep. 2026 May 11;16:21581. doi: 10.1038/s41598-026-53000-w (PMC13351020; doi:10.1038/s41598-026-53000-w)

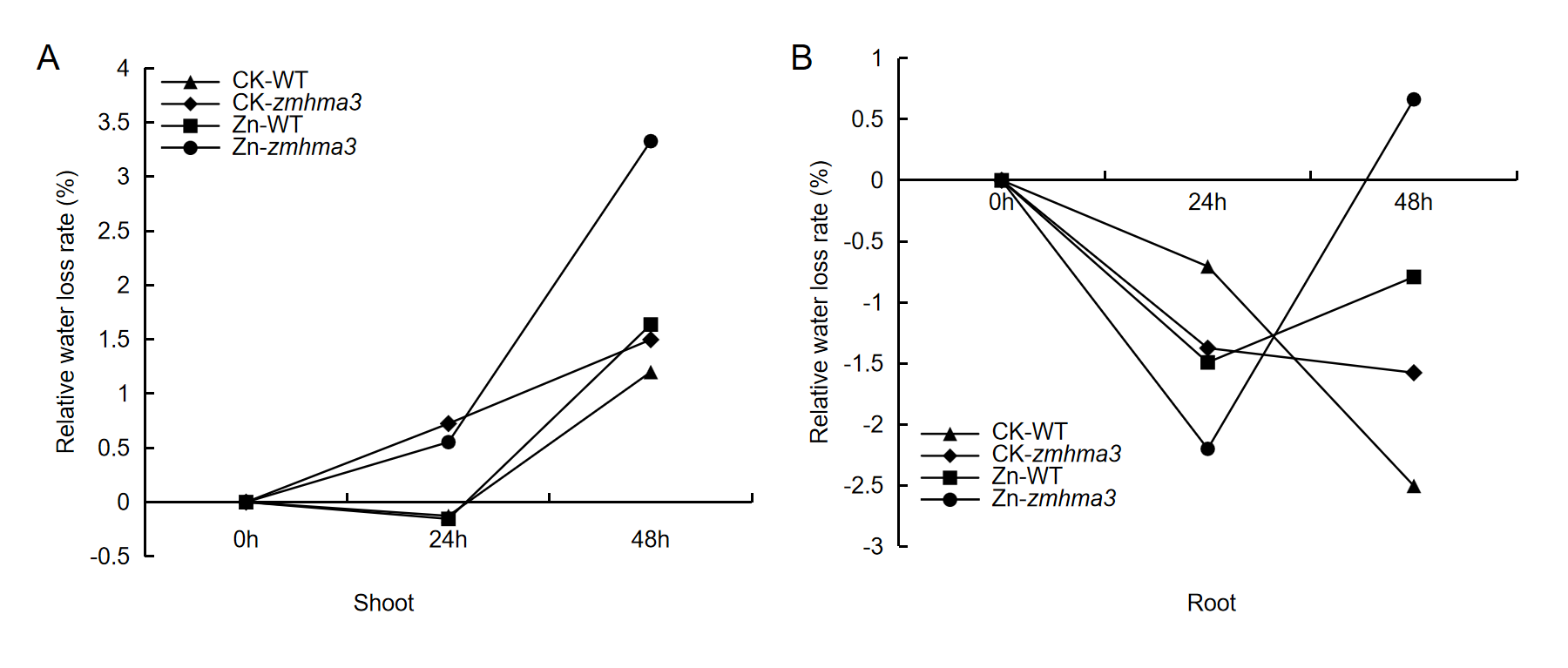

Supplement: Supplementary file 3 — Supplementary Material 3 [file 41598_2026_53000_MOESM3_ESM.tif]
